# Oral antenatal corticosteroids evaluated in fetal sheep

**DOI:** 10.1038/s41390-019-0519-0

**Published:** 2019-07-31

**Authors:** Augusto F. Schmidt, Alan H. Jobe, Paranthaman S. Kannan, James P. Bridges, John P. Newnham, Masatoshi Saito, Haruo Usuda, Yusaku Kumagai, Erin L. Fee, Michael Clarke, Matthew W. Kemp

**Affiliations:** 1 Division of Neonatology and Pulmonary Biology, Cincinnati Children’s Hospital Medical Center, University of Cincinnati, Cincinnati, OH USA; 2 Division of Obstetrics and Gynecology, The University of Western Australia, Perth, WA Australia; 3 Center for Perinatal and Neonatal Medicine, Tohoku University Hospital, Sendai, Miyagi 980-8574 Japan; 4 Metabolomics Australia, Centre for Microscopy, Characterization and Analysis, The University of Western Australia, Perth, WA Australia; 5 School of Veterinary and Life Sciences, Murdoch University, Perth, WA Australia; 6 Present Address: Department of Pediatrics, University of Miami Miller School of Medicine, Miami, FL USA

## Abstract

**Background:**

The use of antenatal corticosteroids (ACS) in low-resource environments is sporadic. Further, drug choice, dose, and route of ACS are not optimized. We report the pharmacokinetics and pharmacodynamics of oral dosing of ACS using a preterm sheep model.

**Methods:**

We measured pharmacokinetics of oral betamethasone-phosphate (Beta-P) and dexamethasone-phosphate (Dex-P) using catheterized pregnant sheep. We compared fetal lung maturation responses of oral Beta-P and Dex-P to the standard treatment with 2 doses of the i.m. mixture of Beta-P and betamethasone-acetate at 2, 5, and 7 days after initiation of ACS.

**Results:**

Oral Dex-P had lower bioavailability than Beta-P, giving a lower maximum maternal and fetal concentration. A single oral dose of 0.33 mg/kg of Beta-P was equivalent to the standard clinical treatment assessed at 2 days; 2 doses of 0.16 mg/kg of oral Beta-P were equivalent to the standard clinical treatment at 7 days as assessed by lung mechanics and gas exchange after preterm delivery and ventilation. In contrast, oral Dex-P was ineffective because of its decreased bioavailability.

**Conclusion:**

Using a sheep model, we demonstrate the use of pharmacokinetics to develop oral dosing strategies for ACS. Oral dosing is feasible and may facilitate access to ACS in low-resource environments.

## Introduction

Antenatal corticosteroids (ACS) are considered by the World Health Organization to be the most effective underutilized treatment available to improve outcomes for fetuses at risk for preterm delivery.^1^ Despite being used clinically for nearly 50 years, the corticosteroid, dose, treatment interval, and route of treatment remain largely unexplored.^2^ While ACS are given to >80% of at-risk pregnancies in advanced clinical care settings, their use is low, and there are questions about efficacy and safety in low medical resource environments where the majority of the mortality from prematurity occurs.^3,4^ An optimal ACS therapy for low-resource environments should expose the mother and fetus to the lowest effective dose for the shortest time using a stable, readily available and inexpensive preparation.^5^

The current standard-of-care treatments may be exposing the fetus to more corticosteroids than necessary.^6,7^ ACS therapy is usually given as 12 mg of a 1:1 mixture of betamethasone-phosphate (Beta-P) and betamethasone-acetate (Beta-Ac) intramuscular (i.m.) at the recognition of preterm labor and a second 12 mg dose 24 h later. Another commonly used treatment is dexamethasone-phosphate (Dex-P) given as four 6 mg maternal i.m. injections given at 12-h intervals. Data from sheep and non-human primate models of pregnancy demonstrate that the slowly soluble Beta-Ac component alone is sufficient to induce fetal lung maturation.^7,8^ The Beta-Ac treatment eliminates the two high maternal and fetal peak Beta levels resulting from the soluble Beta-P in the combined formulation and the four high Dex peaks resulting from the Dex-P treatment. With pharmacokinetic (PK) measurements of Beta-Ac and maternal infusion experiments with Beta-P, we have estimated the fetal blood target range of Beta to cause fetal lung maturation be about 1–4 ng/mL for an exposure duration of about 48 h.^7–9^

The clinical ACS treatments use i.m. maternal injections for drug delivery. However, Beta-P and Dex-P are routinely given orally for multiple indications.^10^ Therefore, we have evaluated the PKs of oral Beta-P and oral Dex-P and tested several dosing schedules for lung maturation in preterm pregnant sheep. These are proof-of-principle experiments for our ultimate goal to optimize ACS treatments in low-resource environments.

## Methods

### Experiments with sheep

The animal experiments were performed in Perth, WA, following review and approval by the Animal Ethics Committee, University of Western Australia and Murdoch University (approval numbers RA/3/100/1378 and R3056/18, respectively). All pregnant sheep were from a single supplier and experiments were performed in July 2017 and July 2018 during the normal delivery season. These experiments used the following corticosteroids: a mixture of 3.9 mg Beta-P and 3.0 mg Beta-Ac per ml (Celestone Chronodose Merck Sharp & Dohme, Macquarie Park, NSW, Australia) for the i.m. maternal standard clinical treatment or oral treatment groups given Beta-P (4.0 mg/ml; Betnesol, Focus Pharmaceuticals, London, UK) or Dex-P (4.0 mg/ml, Hospira Australia, Melbourne, Australia).

### Oral Beta-P and Dex-P PK studies

Beta and Dex concentrations in maternal and fetal plasma were measured as described previously.^11^ Using an aseptic procedure performed under general anesthesia, double lumen catheters were inserted into fetal and maternal jugular veins and secured to the back of the ewe. Ewes were recovered for 24 h before being orally dosed with 0.33 mg/kg Beta-P or 0.33 mg/kg Dex-P in a total volume of 20 mL of normal saline. The ewes were restrained for oral treatments and the drug was administered with a syringe inserted in the ewe’s mouth. The ewes promptly swallowed the drug upon administration. The bioavailability of liquid and tablet formulations of Dex is equivalent.^12^ Maternal and fetal blood samples were drawn and the plasma was recovered and snap frozen until analysis as before.^11^ Beta and Dex plasma standards were run in triplicate for four biological replicates in separate maternal and fetal pairs. Intra-assay coefficient of variation values for mid-range (100 ng/mL per mL plasma) and low-range (12.5 ng/mL) plasma standards were 0.74% and 1.3%, respectively. The assay sensitivity for Dex and Beta is 1 ng/mL with a signal-to-noise ratio of 10:1. Maternal and fetal Beta and Dex concentrations were fitted to a non-compartmental model (extravascular input) using PKSOLVER.^13^

### ACS efficacy studies

These studies were exploratory evaluations of oral dosing of Beta-P and Dex-P performed before the PK data for Dex were available as we assumed comparable PK for Beta and Dex. Ewes with a single fetus received an i.m. injection of 150 mg medroxyprogesterone acetate at 105 days gestational age (Depo-Ralovera; Pfizer, West Ryde, NSW, Australia). The negative saline controls were given maternal i.m. or oral saline. Positive controls were treated with the clinical drug as two doses of Beta-P + Beta-Ac 0.25 mg/kg maternal weight separated by 24 h. Based on the bioavailability of oral Beta-P and Dex-P in other species of about 80% of i.m. administration, we increased our initial oral test doses of Beta-P and Dex-P to 0.33 mg/kg in 20 ml of saline for comparison with the clinical dose.^14^ The treatment groups, doses, and treatment schedules are listed in Table 1. Groups were delivered at 120–123 days gestation at 2, 5, or 7 days after initiation of the treatments to evaluate the lung maturational responses.^7^ The delivery gestation at 120–123 was selected to reliably have fetuses in the early phase of alveolarization and prior to an increase in surfactant (Term = 150 days).^15^

**Table 1:** Description of study groups for oral dosing

Group	Treatment	*N*	Gestational age, days	M/F	BW, kg	Cord blood pH pCO_2_	
Negative control	I.m. or oral saline 2, 5, or 7 days before delivery	25	122 ± 1	14/11	2.7 ± 0.3	7.35 ± 0.06	52 ± 8
Clinical treatment 2 days	0.25 mg/kg Beta-P + Beta-Ac at 0 and 24 h	10	122 ± 0.5	3/7	2.6 ± 0.3	7.35 ± 0.07	50 ± 7
Clinical treatment 5 days	0.25 mg/kg Beta-P + Beta-Ac at 0 and 24 h	8	122 ± 0.8	2/4^a^	2.7 ± 0.2	7.36 ± 0.03	51 ± 5
Clinical treatment 7 days	0.25 mg/kg Beta-P + Beta-Ac at 0 and 24 h	8	122 ± 1.6	2/6	2.9 ± 0.3	7.34 ± 0.04	51 ± 6
Oral Beta-P 2 days	0.33 mg/kg Beta-P at 0 h	9	122 ± 0.5	2/7	2.4 ± 0.1	7.36 ± 0.04	48 ± 6
Oral Beta-P 5 days	0.33 mg/kg Beta-P at 0 h	10	123 ± 0.5	3/7	2.8 ± 0.3	7.36 ± 0.05	50 ± 5
Oral Beta-P 7 days	0.16 mg/kg Beta-P at 0 and 24 h	9	123 ± 0	4/5	2.9 ± 0.3	7.34 ± 0.04	51 ± 4
Oral Dex-P 5 days—1 dose	0.33 mg/kg Dex-P×1 at 0 h	10	121 ± 0.3	6/4	2.7 ± 0.3	7.38 ± 0.03	49 ± 5
Oral Dex-P 5 days—2 doses	0.33 mg/kg Dex-P at 0 and 24 h	10	122 ± 0.5	8/2	2.8 ± 0.2	7.35 ± 0.03	48 ± 3
Oral Dex-P 7 days—3 doses	0.33 mg/kg Dex-P at 0, 12 and 24 h	7	121 ± 0	4/3	2.6 ± 0.1	7.32 ± 0.02	57 ± 3

*Beta-AC* betamethasone-acetate, *Beta-P* betamethasone-phosphate, *BW* birth weight, *Dex-P* dexamethasone-phosphate, *F* female, *I.m.* intramuscular, *M* male

^a^Sex not recorded in two animals

### Ventilation of prematurely delivered lambs

Ewes were given an intravenous bolus of midazolam (0.5 mg/kg) and ketamine (10 mg/kg) and a spinal injection of lidocaine (60 mg in 3 mL) immediately prior to delivery. Lambs were then surgically delivered, given an i.m. injection of ketamine (10 mg/kg), and intubated by tracheostomy. The lambs were ventilated for 30 min using Acutronic Fabian® infant ventilators (Acutronic Medical Systems, Hirzel, Switzerland).^7^ The ventilator delivered heated and humidified 100% oxygen, maximal peak inspiratory pressures of 35–40 cm H_2_O, and a positive end expiratory pressure of 5 cm H_2_O. The respiratory rate was 50 breaths/min, and the inspiratory time was 0.5 s. A maximum tidal volume of 8 mL/kg was targeted by adjusting peak inspiratory pressure only. The investigators responsible for lamb ventilations were blinded to treatment groups.

Arterial blood pH, pO_2_, and pCO_2_ were measured from blood drawn from an umbilical artery catheter. Ventilation data (compliance, tidal volume, peak inspiratory pressure) were recorded at 10, 20, and 30 min.^7^ Ventilation efficiency index (VEI) was calculated as VEI = 3800/[respiratory rate × peak inspiratory pressure − positive end expiratory pressure × pCO_2_ (mm Hg)].^16^ Lambs and ewes were euthanized while anesthetized with an intravenous overdose of sodium pentobarbital. Lamb necropsy was performed as follows: lambs were weighed, and the chest was opened to measure lung compliance with a static pressure–volume curve to 40 cm H_2_O pressure.^7^ The lungs were then removed and weighed and tissue samples were collected. An alveolar lavage was performed on the left lung with samples saved for measurement of saturated phosphatidylcholine (SatPC) as previously reported.^17^

### mRNA measurements for lung maturation

Frozen lung tissues were processed for RNA extraction with TRIzol (Invitrogen, Carlsbad, CA). Reverse transcription reactions were performed using the Verso cDNA kits (Thermo Scientific, Waltham, MA) to produce single-strand cDNA. Gene markers of pulmonary maturation—surfactant protein A (SFTPA), surfactant protein B (SFTPB), sodium channel epithelial 1 alpha subunit (SCNN1A), ATP-binding cassette subfamily A member 3 (ABCA3), and aquaporin 5 (AQP5)—were amplified using sheep-specific primers and Taqman probes (Applied Biosystems, Foster City, CA). Gene expression was normalized for the ribosomal protein 18s mRNA. Data are expressed as fold change relative to negative controls.

### Statistical analysis

Statistical analyses were performed using IBM SPSS for Windows (Armonk, NY). Data were tested for distribution and variance. Mean differences between normally distributed data were tested for significance by two-tailed *t* tests for differences between two groups and with one-way analysis of variance for multiple comparisons (*p* = 0.05). Dunnett’s post-test was used to perform multiple post hoc comparisons. For non-parametric data, Mann–Whitney tests were performed, with significance corrected for *n* comparisons.

## Results

### Pharmacokinetics

**Fig. 1:**
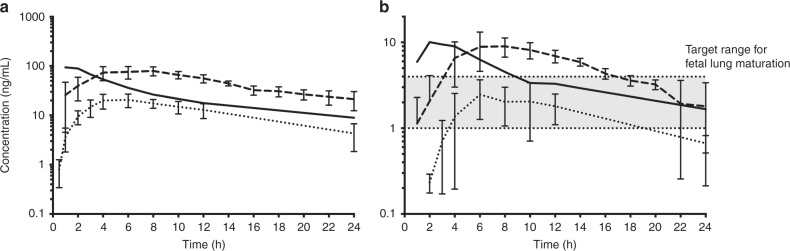
Maternal (**a**) and fetal (**b**) plasma drug levels after single oral dose of 0.33 mg/kg betamethasone-phosphate (Beta-P) (dashed) or 0.33 mg/kg dexamethasone-phosphate (Dex-P) (dotted line) or 0.25 mg/kg of the standard clinical drug (solid line) in pregnant sheep. Oral Dex had decreased absorption compared to oral Beta and resulted in a lower maximal concentration and trough. A single dose of 0.33 mg/kg of oral Beta kept fetal drug levels above the target range (shaded area) for 24 h. The curves for the clinical drug were previously reported^9^

We gave oral doses of 0.33 mg/kg Beta-P and Dex-P to compensate for the presumed decreased bioavailability to about 80% of our i.m. clinical dose of 0.25 mg/kg Beta-P plus Beta-Ac. However, there is no information available about oral absorption or bioavailability of steroids in sheep. The curves in Fig. 1 demonstrate that oral Beta-P slightly reduced the high peak of Beta after the clinical dose and had a comparable maternal exposure over 24 h. Maternal area under the curve (AUC_0–*t*_) values for oral Dex-P and oral Beta-P were 320.6 and 1222.6 ng/mL/h, respectively. Fetal AUC_0–*t*_ values for oral Dex-P and Beta-P were 35.5 and 143.9 ng/mL/h, respectively. The oral Beta-P had a later and slightly lower peak exposure in the fetus, than achieved with clinical dosing, and the Beta level remained in or above the target zone of 1–4 ng/ml for 24 h. Compared to oral Beta dosing, maximum maternal (73.8 vs 20.7 ng/mL) and fetal (8.4 vs 2.5 ng/mL) Dex plasma levels were significantly lower and fell below the target 1–4 ng/mL range much more rapidly (Fig. 1). The maternal and fetal Beta plasma levels from the clinical drug after 6–8 h are from the Beta-Ac because of the more rapid clearance of the Beta-P component of the drug. The half-life of oral Beta was 3.7 h in the ewe and 4.6 h in the fetus, with similar values for Dex. The ratio of maternal to fetal plasma Beta or Dex at maximum concentration was similar (8:1), which is in keeping with data suggesting that placental transfer of Beta and Dex is roughly equivalent.

### Efficacy studies

**Fig. 2:**
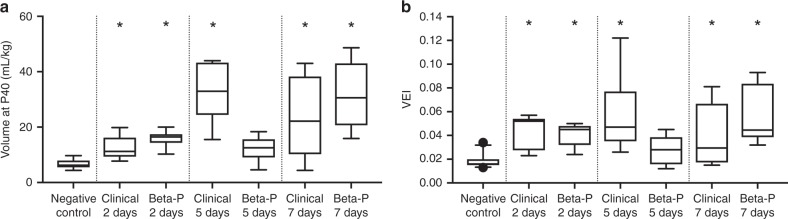
Lung compliance and ventilation efficiency of oral betamethasone (Beta) vs the standard clinical treatment. **a** Lung volume at a pressure of 40 cm H_2_O and **b** ventilation efficiency index (VEI) after oral Beta-phosphate (Beta-P) compared to the standard clinical treatment in preterm sheep. A single dose of 0.33 mg/kg oral Beta-P increased the V40 and VEI similarly to the standard clinical treatment at 2 days but not at 5 days. When given as 2 doses of 24 h apart, the maturational effect of oral Beta-P on the lung persisted up to 7 days. (*p < 0.05)

We randomized pregnant ewes to negative controls with saline or positive controls with the i.m. maternal standard clinical treatments with 0.25 mg/kg Beta-P + Beta-Ac given at 0 and 24 h. Concurrently, ewes were randomized to 0.33 mg/kg oral Dex-P, 0.33 mg/kg or 0.16 mg/kg oral Beta-P for different numbers of treatments and time intervals between treatments. All animal groups were delivered at similar gestational ages with similar birth weights and cord blood pH and pCO_2_ values (Table 1). The ventilator variables at 30 min of ventilation demonstrate improvements relative to negative controls for blood pH and pCO_2_ and with better compliances for the standard clinical treatment for deliveries at 2, 5, and 7 days (Table 2). The pO_2_ values were highly variable. Figure 2 gives values for maximal static lung gas volumes measured at 40 cm H_2_O pressure and for the VEI, a gas exchange measurement that includes pCO_2_, ventilation pressure, and rate. Both these primary outcome measurements for the standard clinical treatment were significantly improved relative to negative controls. While there was variability, the standard clinical treatments were not different at 2, 5, or 7 days. We did not identify differences in response to corticosteroids by gender, although our study was not powered to detect such differences.

**Table 2:** Ventilation variables at 30 min of ventilation

Group	Peak inspiratory pressure, cm H_2_O	Tidal volume, ml/kg	Compliance, ml/kg per cm H_2_O	Arterial blood		
				pH	pCO_2_	pO_2_
Control	38 ± 2	4.4 ± 1.3	0.09 ± 0.03	6.86 ± 0.09	128 ± 23	36 ± 28
Clinical treatment 2 days	35 ± 5	7.2 ± 0.8^a^	0.19 ± 0.08^a^	7.16 ± 0.13^a^	56 ± 23^a^	61 ± 16
Clinical treatment 5 days	35 ± 6	7.7 ± 0.6^a^	0.24 ± 0.06^a^	7.29 ± 0.17^a^	50 ± 15^a^	112 ± 160^a^
Clinical treatment 7 days	33 ± 3^a^	5.6 ± 2.3	0.21 ± 0.11^a^	7.10 ± 0.27^a^	94 ± 51^a^	84 ± 74
Oral Beta-P 2 days	36 ± 4	7.9 ± 0.7^a^	0.21 ± 0.06^a^	7.20 ± 0.11^a^	60 ± 12^a^	112 ± 60^a^
Oral Beta-P 5 days	40 ± 1	6.2 ± 1.8^a^	0.14 ± 0.06^a^	7.07 ± 0.21^a^	92 ± 42^a^	55 ± 34
Oral Beta-P 7 days	32 ± 4^a^	7.2 ± 0.7^a^	0.28 ± 0.07^a^	7.24 ± 0.10^a^	56 ± 15^a^	175 ± 160^a^
Oral Dex-P 5 days × 1 dose	40 ± 0	5.5 ± 1.4	0.11 ± 0.06	7.01 ± 0.15^a^	104 ± 38	36 ± 16
Oral Dex-P 5 days × 2 doses	36 ± 4	7.2 ± 1.6^a^	0.20 ± 0.07^a^	7.18 ± 0.17^a^	62 ± 19^a^	61 ± 77
Oral Dex-P 7 days × 3 doses	35 ± 0^a^	3.9 ± 2.0	0.13 ± 0.07	6.91 ± 0.19	126 ± 41	47 ± 37

*Beta-AC* betamethasone-acetate, *Beta-P* betamethasone-phosphate, *Dex-P* dexamethasone-phosphate

^a^*p* < 0.05 relative to negative control

The oral Beta-P treatment given as 1 dose and evaluated at 2 days had similar improvements in compliance, pCO_2_, V40, and VEI as did the standard clinical treatment at 2 days (Table 2, Fig. 2). The 1 dose Beta-P effects were not significantly different from negative controls at 5 days. In contrast, a 2-dose oral Beta-P treatment separated by 24 h with 0.16 mg/kg Beta-P was qualitatively the best treatment with the highest compliance, V40, and VEI measurements at 7 days that were not significantly different from the standard clinical treatment with 2 doses at 7 days.

**Fig. 3:**
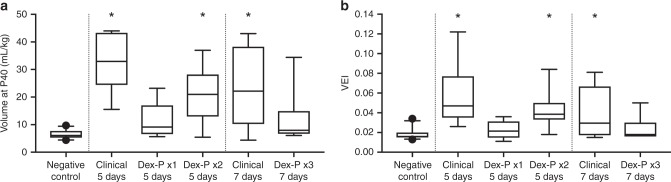
Lung compliance and ventilation efficiency of oral dexamethasone (Dex) vs the standard clinical treatment. **a** Lung volume at a pressure of 40 cm H_2_O and **b** ventilation efficiency index (VEI). A single dose of oral Dex was not as effective as the standard clinical treatment in improving lung compliance and ventilation efficiency, and the effect was improved when given as 2 doses of oral Dex 24 h apart. The administration of 3 doses of oral Dex 12 h apart was not effective in producing a lasting effect on lung maturation to 7 days. (*p < 0.05)

The single oral Dex-P treatment evaluated at 5 days was not different from negative controls, indicating no efficacy (Fig. 3). Two doses of Dex-P given at 24 h increased all the blood gas ventilation variables significantly relative to negative controls to values that approached and were not different from the standard clinical treatment. In contrast, a 3-dose treatment at 12-h intervals and evaluated at 7 days had no significant indicators of improved lung function relative to negative controls. The increased dosing did not result in a durable Dex-P effect at 7 days.

### Other indications of maturation

**Fig. 4:**
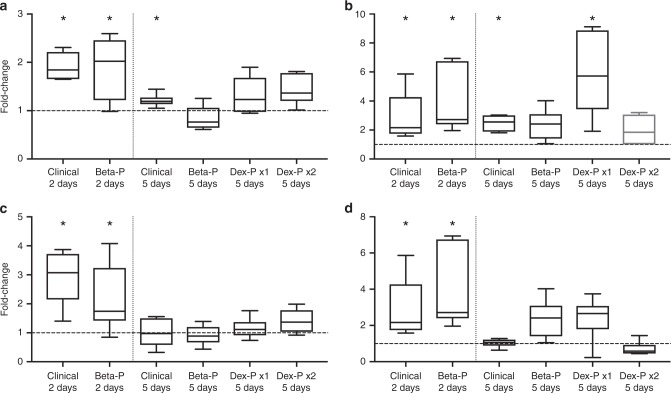
mRNA levels of the select genes associated with lung maturation: **a** Surfactant protein B (SFTPB); **b** ATP-binding cassette subfamily 3 (ABCA3); **c** sodium channel epithelial 1 alpha subunit (SCNN1A); **d** aquaporin-5 (AQP5); (*n* = 6–7 animals/group). (*p < 0.05)

**Fig. 5:**
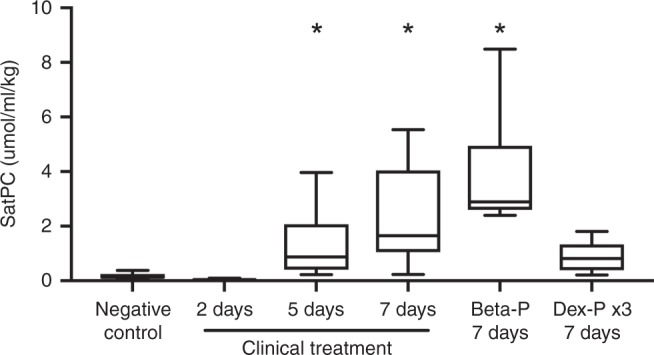
Saturated phosphatidylcholine (SatPC) concentration in the bronchoalveolar lavage fluid after treatment of preterm sheep with oral or intramuscular corticosteroids. Oral betamethasone-phosphate increased the SatPC concentration equivalently to the standard clinical treatment, while oral dexamethasone did not; *n* = 6–7 animals/group (*p < 0.05)

In fetal sheep, lung mechanics improve with effective ACS within 24 h. While mRNA changes are detectable early after administration of ACS and return to levels similar to negative controls by about 5 days after treatment, SatPC does not increase until after about 4 days.^18^ We analyzed mRNA levels by real-time PCR at 2 and 5 days after ACS (Fig. 4). At 2 days, oral Beta and the standard clinical treatment increased the mRNA levels of SFTPB, SCNN1A, ABCA3, and AQP5. At 5 days, gene levels of maturation markers had returned to levels similar to negative controls, except for the levels of SFTPB and ABCA3 after the standard clinical treatment and ABCA3 after oral Dex-P x1. We measured SatPC at 7 days after ACS. The standard clinical treatment and the two doses of Beta-P treatment increased Sat PC in the alveolar lavages (Fig. 5). In contrast, three doses of Dex-P given at 12-h intervals did not increase SatPC.

## Discussion

As proof of principle, oral dosing of ACS induced fetal lung maturation equivalently to the two-dose standard clinical treatment with Beta-P+Beta-Ac. However, oral Dex-P and Beta-P differ strikingly in their absorption and bioavailability in sheep. Peak maternal and fetal blood levels were measured at about 6–8 h in sheep. In contrast, oral Dex-P yields shorter times of <2 h to peak levels in healthy adults and in women being treated with ACS for the risk of preterm delivery.^12,14^ Oral and i.m. Dex-P yield similar biological half-life values and high estimates of bioavailability ranging from 65% to 100%.^14,19^ There is very little information about Beta-P in pregnant women beyond some measurements with the Beta-P+Beta-Ac combination.^20–22^ In sheep for equivalent oral doses of 0.33 mg/kg, peak maternal plasma levels of Dex-P were approximately only 33% of the values for oral Beta-P demonstrating greatly decreased bioavailability of Dex than Beta in sheep after oral administration. The PKs of Beta and Dex clearly differ for sheep and humans with decreased bioavailability of oral Dex-P in sheep. Nevertheless, we were able to demonstrate the principles for oral dosing of ACS. PK studies of Beta-P and Dex-P comparing i.m. and oral formulations in women are currently being evaluated (ClinicalTrials.gov Identifier: NCT03668860).

We performed the PK studies of fetal lung responses to ACS based on the PK information that we had for Beta-P, and we incorrectly assumed that Dex-P levels would be comparable. We evaluated a high-dose estimate of the drugs given orally based on 80% bioavailability as a representative value in humans.^14^ We previously reported that fetal sheep responded to ACS blood levels in the range of 1–4 ng/mL based on studies with Beta-Ac and maternal infusions of Beta-P to target that fetal blood level.^9,11^ We were also concerned about the durability of the ACS response if fetal drug level were not maintained for at least 48 h. As the ratio of the fetal to maternal plasma levels are about 0.1 for Dex or Beta in sheep, relatively large maternal doses need to be given to achieve adequate fetal plasma drug levels. In contrast, the fetal-to-maternal blood level ratios for humans is about 0.5,^21^ suggesting that lower oral doses should be sufficient for lung maturation.

Our results support the validity of the estimated minimal fetal exposure to >1 ng/mL for a lung maturation stimulus. We used the standard clinical dosing with two doses of 0.25 mg/kg Beta-P+Beta-Ac as the presumed gold standard for a Beta exposure that will cause fetal lung maturation. The previously reported maternal and fetal Beta levels cause excessive maternal and fetal drug exposures for about 8 h, and the second dose should keep Beta levels in the fetus >1 ng/mL for 48 h (Fig. 1).^9,11,23^ Our dose of 0.33 mg/kg oral Beta-P resulted in excessive fetal exposures between 4 and 16 h. A single oral Beta treatment induced lung maturation comparable to the standard clinical 2-dose treatment at 2 but not at 5 days, presumably because the single dose was not sufficient for durability to 5 days. However, we tested a 2-dose 24-h treatment interval with a lower oral dose of 0.16 mg/kg, which should keep fetal Beta levels >1 ng/ml for at least 48 h and cause lung maturation at 7 days. In contrast, 1 or 2 doses of oral Dex at 0.33 mg/kg caused minimal lung maturational responses at 5 days as did 3 doses at 7 days as anticipated based on the PKs of Dex in sheep. These detailed PK and pharmacodynamic assessments for ACS cannot be performed with precision in women at risk of preterm delivery. The early mRNA changes of maturation markers after oral Beta were similar to the standard clinical treatment and consistent with the physiological observation of mechanical lung improvement with the oral Beta treatment and the increase in SatPC concentration in the bronchoalveolar fluid at 7 days.

There is minimal clinical information about oral dosing for ACS. Egerman and colleagues reported a PK study with oral Dex-P and used that information for a clinical study that was stopped early for adverse fetal effects with oral treatment.^14,24^ The authors commented that the complications of neonatal sepsis and intraventricular hemorrhage were much lower than anticipated in the maternal i.m. Dex group relative to the oral Dex-P and that further research on oral ACS was warranted. There is no good explanation about why oral vs i.m. dosing should differ in lung maturational response if comparable fetal exposures are achieved.

The studies demonstrate that drug characteristics, dose, and treatment intervals are critical for durable maturational responses in sheep. Multiple drugs, doses, and dosing intervals are used in humans without consideration of these variables or validation of clinical responses. These studies together with our previous evaluation of i.m. and infusions of Beta provide insight about how ACS therapy can be optimized. Our proof-of-concept experiments in sheep demonstrate that oral dosing for ACS is feasible.
